# ROS: Executioner of regulating cell death in spinal cord injury

**DOI:** 10.3389/fimmu.2024.1330678

**Published:** 2024-01-23

**Authors:** Zhaoyang Yin, Bowen Wan, Ge Gong, Jian Yin

**Affiliations:** ^1^Department of Orthopedics, the Affiliated Lianyungang Hospital of Xuzhou Medical University (The First People’s Hospital of Lianyungang), Lianyungang, China; ^2^Department of Orthopedics, Northern Jiangsu People’s Hospital Affiliated to Yangzhou University/Clinical Medical College, Yangzhou University, Yangzhou, China; ^3^Department of Geriatrics, Jinling Hospital, Affiliated Hospital of Medical School, Nanjing University, Nanjing, China; ^4^Department of Orthopedics, the Affiliated Jiangning Hospital with Nanjing Medical University, Nanjing, China; ^5^Department of Orthopedics, Jiangning Clinical Teaching Hospitals of Jiangsu Vocational College of Medicine, Nanjing, China

**Keywords:** spinal cord injury, ROS, apoptosis, autophagy, ferroptosis, pyroptosis

## Abstract

The damage to the central nervous system and dysfunction of the body caused by spinal cord injury (SCI) are extremely severe. The pathological process of SCI is accompanied by inflammation and injury to nerve cells. Current evidence suggests that oxidative stress, resulting from an increase in the production of reactive oxygen species (ROS) and an imbalance in its clearance, plays a significant role in the secondary damage during SCI. The transcription factor nuclear factor erythroid 2-related factor 2 (Nrf2) is a crucial regulatory molecule for cellular redox. This review summarizes recent advancements in the regulation of ROS-Nrf2 signaling and focuses on the interaction between ROS and the regulation of different modes of neuronal cell death after SCI, such as apoptosis, autophagy, pyroptosis, and ferroptosis. Furthermore, we highlight the pathways through which materials science, including exosomes, hydrogels, and nanomaterials, can alleviate SCI by modulating ROS production and clearance. This review provides valuable insights and directions for reducing neuronal cell death and alleviating SCI through the regulation of ROS and oxidative stress.

## 1 Introduction

Spinal cord injury (SCI) is a prevalent form of central nervous system injury that leads to motor, sensory, and autonomic dysfunction. In the United States alone, the annual incidence of spinal cord injuries ranges from 12,000 to 20,000 (1). According to a study conducted by the Global Burden of Disease in 2016, there are approximately 93,500 new cases of SCI worldwide each year, with a total of 27 million existing cases (2). The pathophysiological process of SCI primarily consists of two stages: primary injury and secondary injury. Primary injury involves the physical forces that occur during the initial traumatic event, such as compression, tearing, shearing, and stretching (3). Secondary injury refers to the continuation of primary injury and is characterized by vasogenic edema, as mentioned above, mitochondrial dysfunction, and excessive production of reactive oxygen species (ROS) (4, 5). The overproduction of ROS plays a crucial role in the secondary injury process, further exacerbating the inflammatory response and various regulatory cell death (RCD) pathways (6). Due to the complexity of SCI’s pathophysiology, there is currently no satisfactory clinical intervention method that can significantly improve neurological function. Effectively mitigating the extensive cell death caused by secondary injury is the key to preserving the neurological function of the damaged spinal cord.

The current understanding of secondary injury mechanisms in SCI primarily involves oxidative stress and nitrosative stress (7). Oxidative and nitrosative stress are thought to be an imbalance between the presence of high levels of ROS and reactive nitrogen species (RNS), and antioxidant defense mechanisms (8). Oxidative stress response can be considered a double-edged sword. In the early stages, low levels of oxidative stress can provide some protection to damaged tissues and cells (9, 10), However, sustained high levels of oxidative stress can worsen inflammation and cell death (11, 12). During cellular metabolic activities, free radicals are generated and can be counteracted by endogenous antioxidants, maintaining redox balance. The primary molecule involved in oxidative stress is the superoxide O2·^-^ radical, which is produced when electrons in O2 molecules are reduced (13). Interestingly, this radical can function both as an oxidizing agent and a reducing agent. ROS and RNS, which are highly reactive free radicals, can cause damage to cell membranes, proteins, and DNA, triggering inflammatory cascade events (14). Oxygen free radicals are particularly harmful to polyunsaturated fatty acids, leading to lipid peroxidation (15). There is increasing evidence suggesting that oxidative stress, induced by excessive ROS, plays a critical role in the secondary injury of spinal cord injury (SCI) (9, 16, 17). ROS include superoxide anion radical (O_2_-), hydrogen peroxide (H_2_O_2_), hydroxyl radical (·OH), and peroxynitrite (18). These ROS are produced through enzymatic and non-enzymatic pathways during normal metabolic processes (19). While natural ROS scavenging enzymes like superoxide dismutase (SOD), catalase (CAT), and glutathione peroxidase (GPx) have limited stability, availability, and high cost, current research focuses on developing exogenous ROS scavengers. Additionally, researchers are continuously updating material systems to address challenges related to antioxidant loading and delivery. This article provides a comprehensive review of the role of reactive ROS in apoptosis, autophagy, pyroptosis, and ferroptosis during SCI. It also explores the potential crosstalk mechanisms involved, aiming to offer new insights and directions for mitigating nerve damage through the elimination of excess ROS.

## 2 ROS in apoptosis

Signal regulation between ROS and apoptosis is a highly significant area of research in life sciences. In addition to H_2_O_2_, oxidized low-density lipoprotein and peroxynitrite free radicals have been found to induce various types of apoptosis (20). Antioxidants play a role in suppressing signaling cascades that lead to apoptosis (21). Some studies have shown that depletion of reduced glutathione is associated with the initiation of apoptosis (22, 23). Recently, there has been extensive research on the control of SCI through microRNA (miRNA) modulation. MiRNAs are non-coding small RNA molecules composed of 20-24 nucleotides, and they actively participate in the regulation of oxidative stress (24, 25). Two independent studies have demonstrated similar modes of action, where H2O2 and LPS inhibit the expression of MiR-495 and MiR−122−5p in neural cells respectively, resulting in reduced ROS production and cell apoptosis. Mechanistically, MiR-495 downregulates the expression of STAT3, while MiR-122-5p represses CPEB1, targeting the PI3K/AKT signaling pathway (26, 27). Another study on traditional Chinese medicine suggests that calycosin promotes the expression level of p-AKT by up-regulating heat shock protein 90 (HSP90), while inhibiting the expression levels of p-ASK1 (pThr845) and p-p38, thereby alleviating ROS, oxidative stress, and apoptosis in neurons (28). In contrast to what was mentioned above (26), the role of AKT phosphorylation in promoting or inhibiting ROS generation and apoptosis is still controversial. This discrepancy could potentially be attributed to differences in phosphorylation sites.

The reduction of mitochondrial membrane potential (MMP), intracellular calcium overload, opening of mitochondrial permeability transition pore (mPTP), and generation of reactive oxygen species (ROS) during spinal cord ischemia-reperfusion injury (SCIRI) lead to mitochondrial dysfunction and apoptosis of neural cells (29, 30). In the SCIRI or oxygen glucose deprivation/reperfusion (OGD/R) model, polydatatin promotes the nuclear import of nuclear factor erythroid 2-related factor 2 (Nrf2) to bind to antioxidant response elements (ARE) by accelerating the dissociation of Kelch-like ECH-associated protein 1 (Keap1)/Nrf2 complex. This enhances the antioxidant activity of spinal motor neurons (SMNs), which is beneficial for increasing MMP, clearing ROS, and restoring mitochondrial function. By reversing damaged mitochondria, polydatin inhibits the apoptosis of SMNs through the classic Bcl-2/cytochrome c pathway (29). Ischemia-reperfusion-induced changes in cellular metabolism lead to inflammation-related epigenetic modifications, post-transcriptional regulation, and post-translational modifications of proteins (31–33). Phosphoglycerate kinase 1 (PGK1) is a key enzyme involved in ATP generation during glycolysis in cellular metabolism and is linked to oxidative stress through the Keap1-Nrf2 pathway (34–36). Dimerization of Keap1 inhibits Nrf2 ubiquitination, promoting Nrf2 nuclear import and antioxidant effects (37–39). A recent study suggested that G-protein-coupled receptor (GPCR) kinase 2-interacting protein-1 (Git1), a GTPase-activating protein, regulates the phosphorylation of PGK1 at S203 in 293T cells under OGD/R conditions, thus affecting Keap1-Nrf2 signaling and oxidative stress (40). Git1 in neurons enhances Nrf2 signaling by promoting Keap1 dimerization to reduce reperfusion-induced ROS levels, providing a potential target for SCIRI treatment.

**Figure 1:**
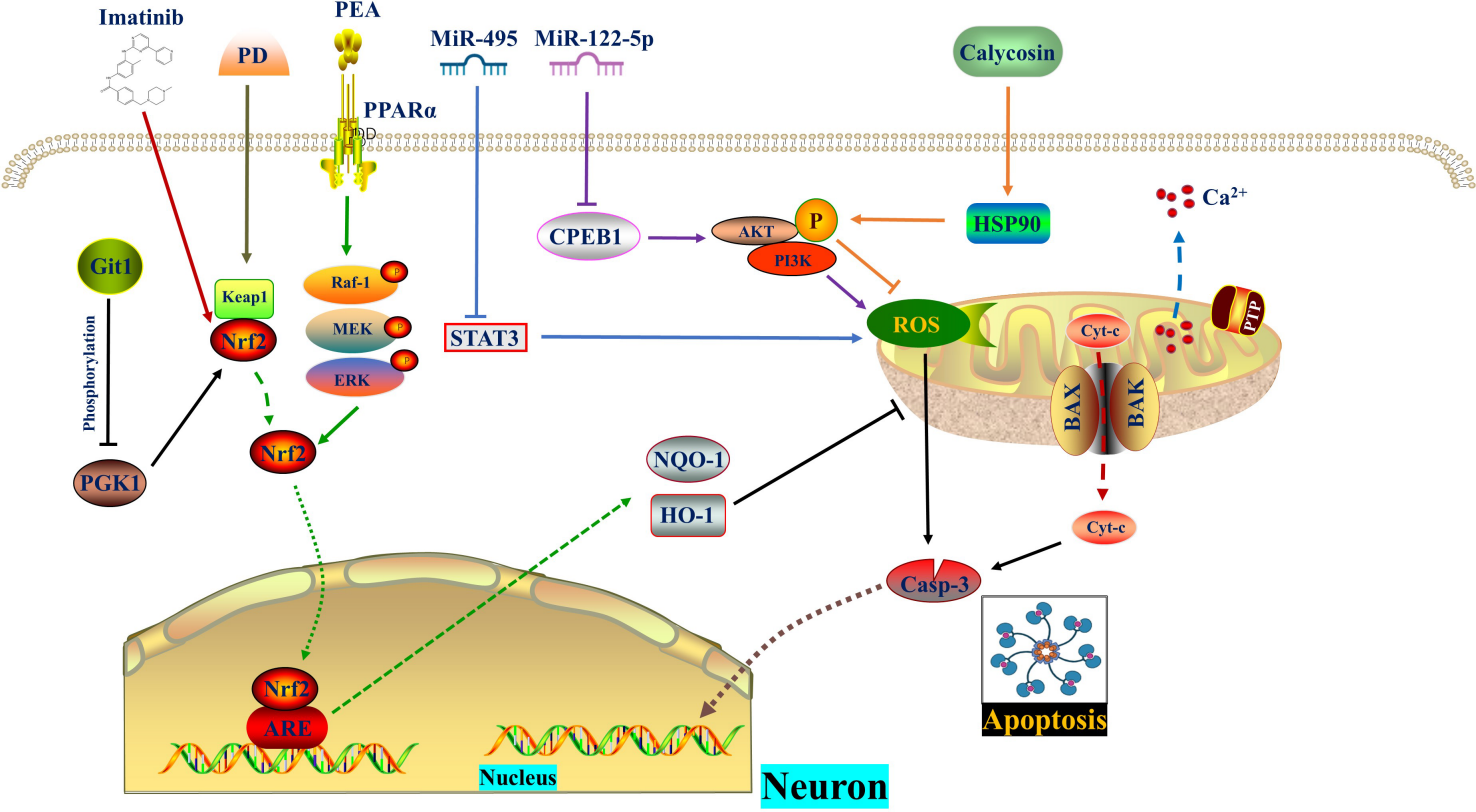
ROS in apoptosis. MiR-495 and MiR−122−5p reduce ROS level and apoptosis by inhibiting STAT3 and CPEB1, respectively. Calycosin promotes AKT phosphorylation by increasing HSP90 expression, thereby diminishing ROS generation and apoptosis. PD promotes the dissociation of Nrf2 from Keap1. Subsequently, Nrf2 enters the nucleus and binds to AREs, thereby enhancing cellular oxidation resistance and reversing mitochondrial damage as well as apoptosis. Git1 regulates PGK1 phosphorylation to counteract ROS through the Keap1/Nrf2 axis. Imatinib relieves oxidative stress and apoptosis by activating Nrf2. The PPARα agonist PEA activates Nrf2 through phosphorylation of the Raf‐1/MEK/ERK pathway, thereby inhibiting subsequent oxidative stress and apoptosis. CPEB1, Cytoplasmic polyadenylation element binding protein 1; ERK, Extracellular signal-regulated kinase; Git1, G-protein-coupled receptor (GPCR) kinase 2-interacting protein-1; HSP90, Heat shock protein 90; IR, Ischemia reperfusion; MEK, Mitogen-activated protein kinase; PD, Polydatin; PEA, Palmitoylethanolamide, a peroxisome proliferator-activated receptor alpha (PPARα) agonist; PGK1, Phosphoglycerate kinase 1; PPARα, Peroxisome proliferator-activated receptor α; Raf-1, V-raf-1 murine leukemia viral oncogene homolog 1; ROS, Reactive oxygen species.

Peng Zou et al. (41) observed a significant upregulation of the expression of excision repair cross-complementing group 6 (ERCC6, also known as CSB) in SCI mice and LPS-induced microglia. ERCC6 is a DNA-binding protein with ATPase activity that inhibits apoptosis and contributes to tumor proliferation (42, 43). Silencing ERCC6 not only hindered the activation of astrocytes and microglia, thereby reducing the inflammatory response, but also prevented microglial apoptosis and alleviated neuronal damage. Mechanistically, ERCC6 silencing reduced cellular oxidative stress by decreasing the expression of 4-HNE, Nrf2, and Keap1, as well as reducing excessive ROS accumulation in the spinal cord after SCI (41). Imatinib, a tyrosine kinase inhibitor, promoted the expression of HO-1 and SOD by activating Nrf2, thus providing resistance against oxidative stress and apoptosis (44). Additionally, imatinib, an anti-tumor drug used for chronic lymphocytic leukemia, can protect the blood-brain barrier and alleviate inflammation (45). The V-raf-1 murine leukemia viral oncogene homolog 1 (Raf-1)/mitogen-activated protein kinase (MEK)/extracellular signal-regulated kinase (Erk) pathway, a classic MAPK pathway, plays a role in cell proliferation, differentiation, inflammatory response, and apoptosis. Activation of Raf kinase leads to the activation of downstream MEK 1/2, enabling ERK1/2 to translocate from the cytoplasm to the nucleus and exert biological effects (46–49). Research by Haocong Zhang et al. (50) demonstrated that palmitoylethanolamide (PEA), an agonist of peroxisome proliferator-activated receptor α (PPARα), activates Nrf2 through phosphorylation of the Raf-1/MEK/ERK pathway, thereby inhibiting oxidative stress, inflammatory response, apoptosis, and promoting motor function recovery in SCI rats (**Figure 1**) (**Table 1**).

**Table 1:** Experimental study on ROS as a therapeutic target in SCI - molecular mechanism.

Research Topics	Therapeutic methods	Animal	Cell type	Animal model	In vitro cell model	Molecular mechanism	Ref
Apoptosis	MiR-495	*Not implemented*	Primary neurons of the spinal cord	*Not implemented*	Stimulated by H_2_O_2_	miR-495 ameliorates ROS and SCNs apoptosis by inhibiting STAT3 expression.	(25) Yunfeng Qiu
Apoptosis	MiR−122−5p	C57B/L6 mice	SH-SY5Y	*Not mentioned*	Stimulated by LPS (1 μg/mL, 24 h)	MiR-122-5p impedes PI3K/AKT phosphorylation by reducing CPEB1 expression, thereby inhibiting ROS generation and apoptosis.	(26) Zijian Wei
Apoptosis	Calycosin	SD rats	PC12 cells	30 g forceps on T8-10 spinal cord for 60s	Stimulated by H_2_O_2_ (12 h)	Calycosin promotes the expression of p-Akt and inhibits the expression of p-ASK1 (pThr845)/p-p38 by up-regulating HSP90, thereby alleviating neuronal ROS and apoptosis.	(27) Mingdong Li
Apoptosis Nrf2	Polydatin	C57B/L6 mice	Primary SMNs	Clamping between the distal thoracic aorta and left subclavian artery for 5 minutes	OGD/R	Polydatatin reverses mitochondrial damage in SMNs through Keap1/Nrf2/ARE pathway and inhibits apoptosis through Bcl-2/Cyt-c pathway.	(28) Jiheng Zhan
Apoptosis Nrf2	Git1	C57B/L6 mice	Mice or HEK293T cells	Abdominal aorta occlusion for 60 minutes	OGD/R	Git1 alleviates ROS levels and neuronal loss in SCIRI by regulating the Keap1-Nrf2 signaling pathway through phosphorylation of PGK1.	(38) Tao Xu
Apoptosis Nrf2	Imatinib	SD rats	*Not implemented*	An impacting rod hits T10 spinal cord from 1.25 cm height	*Not implemented*	Imatinib relieves oxidative stress and apoptosis by activating Nrf2 pathway.	(42) Limin Liu
Nrf2	PPARα agonist	SD rats	*Not implemented*	A 5 g weight hits T11 spinal cord from 10 cm height	*Not implemented*	PEA activates Nrf2 by phosphorylating the Raf‐1/MEK/ERK pathway, thereby inhibiting subsequent oxidative stress and apoptosis.	(48) Haocong Zhang
Apoptosis Nrf2	ERCC6	C57B/L6 mice	Murine BV2 microglial cells	A 10 g weight hits T9-10 spinal cord from 1.5 cm height	Stimulated by LPS (0, 25, 50, and 100 ng/ml)	ERCC6 silencing, on the one hand, inhibits microglial activation and apoptosis, and on the other hand, inhibits the expression of Nrf2, Keap1 and ROS accumulation.	(39) Peng Zou
Autophagy Mitophagy Parthanatos	Zinc	C57B/L6 mice	VSC4.1 cells	A 12.5 g weight hits T9-10 spinal cord from 1.5 cm height	Stimulated by H_2_O_2_ (80 μmol/L, 3 h)	Zinc interacts with SIRT3 to alleviate Parthanatos by targeting SOD2 via acetylation and promoting mitophagy to eliminate ROS, respectively.	(51) Dingyuan Jiang
Autophagy ERS	TFE3	C57B/L6 mice	PC12 cells	A 10 g weight hits T9-10 spinal cord from 1.5 cm height	Stimulated by TBHP	TFE3 promotes autophagic lysosomal activity and inhibits ROS accumulation to restore autophagic flux, thereby reducing ERS-induced apoptosis.	(52) Kailiang Zhou
Pyroptosis	Kaempferol	SD rats	Murine BV2 microglial cells	A 1.5 mm diameter impactor produces 2.0 mm displacement at C5 at 500 mm/s	Stimulated by LPS (1 μg/mL) and ATP (5 mM Sigma) for 2 h	Kaempferol inhibits microglial activation and NLRP3-mediated pyroptosis by hindering NOX4-ROS-MAPKs-NF-κB signaling pathway^*^.	(53) Zhongyuan Liu
Pyroptosis	AOPPs	SD rats	Murine BV2 microglial cells	The striker impacts the C5 at 500 mm/s to trigger a displacement of 2.2 mm	Stimulated by AOPPs-MSA	AOPPs activate microglia and NLRP3-mediated pyroptosis through the NOX4-ROS-MAPKs-NF-κB signaling pathway, and this process can be reversed by Apocynin^*.^.	(54) Zhongyuan Liu
Pyroptosis Apoptosis	Hv1	C57B/L6 mice	PC12 cells	A 5 g weight hits T10 spinal cord from 1.1 cm height	OGD/R	Hv1 deficiency inhibits neuronal NLRP3-mediated pyroptosis and apoptosis by reducing microglial ROS production.	(55) Xuefei Li
Pyroptosis Autophagy Mitophagy	BA	C57B/L6 mice	*Not implemented*	A 15 g weight hits T11-12 spinal cord from 1.5 cm height	*Not implemented*	BA activates macroautophagy through the AMPK-mTOR-TFEB pathway to enhance mitophagy, thereby reducing ROS accumulation and inhibiting pyroptosis.	(56) Chenyu Wu
Pyroptosis Autophagy	Piperine	C57B/L6 mice	*Not implemented*	15 g forceps on T8 spinal cord for 60s	*Not implemented*	Piperine reduces ROS formation and NLRP3/GSDMD-mediated pyroptosis by activating autophagy.	(57) Haojie Zhang
Nrf2	Melatonin	C57B/L6 mice	PC12 cells	A 12.5 g weight hits T9-10 spinal cord from 2.0 cm height	Stimulated by H_2_O_2_ (20µMol) and ATP (5 mmol/L) for 6 h	Melatonin diminishes NLRP3 inflammasome, ROS, and mitochondrial dysfunction through the Nrf2/ARE pathway.	(58) Haoyu Wang
Ferroptosis	DFO, Fer-1 NAC, Minocycline, L-NAME	SD rats	Primary cortical neurons	50 g forceps on T9 spinal cord for 60s	Stimulated by FAC	DFO, Fer-1 and NAC inhibited neuronal ferroptosis, respectively. Minocycline and L-NAME reverse neuronal ferroptosis by reducing NO released from microglia.	(59) Zhou Feng
Ferroptosis	Trehalose	Mice	Hippocampal tissue of C57B/L6 mice	A 10 g weight hits T10 spinal cord from 2.5 mm^**^ height	Stimulated by Erastin (5 μM, 12 h)	Trehalose reduces the generation of ROS, thereby inhibiting neuronal ferroptosis by activating the NRF2/HO-1 pathway.	(60) Fangyi Gong
Ferroptosis	AS-IV	*Not implemented*	PC12 cells	*Not implemented*	Stimulated by H_2_O_2_ (300 μmol/L)	AS-IV inhibits ferroptosis by promoting TFEB expression.	(61) Yifei Zhou
Ferroptosis	Hepcidin	Wistar rats	Primary OPCs	A 20 g weight hits T10 spinal cord from 3.0 cm height	Stimulated by FeCl_3_ (20µM)	Hepcidin inhibits ferroptosis of OPCs by reducing ROS production and DMT1/TfR1 expression.	(62) Shengli Hu
Ferroptosis	Ferrostatin-1	Wistar rats	Primary OPCs	A 20 g weight hits T10 spinal cord from 3.0 cm height	Stimulated by FeCl_3_ (20µM)	Ferrostatin-1 inhibits ferroptosis in OPCs by reducing ROS generation and IREB2/PTGS2 expression.	(63) Shengli Hu

^*^ The MAPKs here refer to p-p38 and p-JNK, but do not include p-ERK1/2.

^**^ The author described it as 2.5mm in the original article, but we believe it should be 2.5cm.

AOPPs, Advanced oxidation protein products; AS-IV, Astragaloside IV; BA, Betulinic acid; CPEB1, Cytoplasmic polyadenylation element binding protein 1; DFO, Deferoxamine, an iron chelator; ERK, Extracellular signal-regulated kinase; ERS, Endoplasmic reticulum stress; FAC, Ferric ammonium citrate; Fer-1, Ferrostatin-1, a ferroptosis inhibitor; Git1, G-protein-coupled receptor (GPCR) kinase 2-interacting protein-1; HSP90, Heat shock protein 90; Hv1, The microglial voltagegated proton channel; LPS, Lipopolysaccharide; MEK, Mitogen-activated protein kinase; NAC, N-acetylcysteine, a ROS inhibitor; NO, Nitric oxide; NADPH, Nicotinamide adenine dinucleotide phosphate hydrogen; NOX4, NADPH oxidase 2; OGD/R, Oxygen and glucose deprivation and reperfusion; OPCs, oligodendrocyte progenitor cells; PARP-1, poly ADP-ribose polymerase-1; PEA, Palmitoylethanolamide, a peroxisome proliferator-activated receptor alpha (PPARα) agonist; PGK1, Phosphoglycerate kinase 1; PLAS, Poly (lipoic acidco-sodium lipoate); PPARα, Peroxisome proliferator-activated receptor α; Raf-1, V-raf-1 murine leukemia viral oncogene homolog 1; SCIRI, Spinal cord ischemia-reperfusion (IR) injury; SCNs, Spinal cord neurons; SMNs, Spinal motor neurons; SIRT3, Sirtuin 3; SOD2, Superoxide dismutase 2; TBHP, Tert-butyl hydroperoxide; TFE3, Transcription factor E3.

## 3 ROS in autophagy

Autophagy is a unique phenomenon in eukaryotic cells that involves the degradation of damaged proteins and organelles for recycling through the lysosomal pathway. It is important to note that autophagy has both positive and negative effects (64, 65). There are three categories of autophagy: macroautophagy, chaperone-mediated autophagy, and microautophagy. When we refer to autophagy, we usually mean macroautophagy (66). The occurrence of autophagy is regulated by a series of autophagy protein families called ATG proteins, which are structurally and functionally conserved (67). Autophagy is induced in the body by factors such as deprivation of amino acids, oxygen, and sugar, low insulin levels, and reduced ATP levels. In the case of SCI, secondary pathological changes like hypoxia, hemorrhage, and edema lead to mitochondrial dysfunction and increased activity of ROS-generating enzymes, resulting in the production of large amounts of ROS (68). ROS then oxidizes unsaturated fatty acids in the lysosomal membrane, causing proteolytic enzyme extravasation and disruption of lysosomal function, which in turn blocks autophagic flux in SCI (69, 70). Impaired autophagy has been shown to inhibit ubiquitination, weaken mitochondrial function, and accumulate ROS, thereby disrupting cellular homeostasis (66). On the other hand, excessive autophagy can lead to autophagic death of spinal cord nerve cells (71, 72). Therefore, it is crucial to strictly control the degree of autophagy to maintain optimal levels (73). Mitochondria, which are not only essential for cellular energy metabolism and mitochondrial homeostasis, but also the main organelles that generate ROS after SCI, play a significant role. Interestingly, ROS can worsen mitochondrial damage and DNA oxidation, creating a vicious cycle that ultimately leads to cell death (56, 74, 75). Mitophagy, a selective autophagic degradation process specifically targeting damaged mitochondria, helps maintain mitochondrial homeostasis and cell survival by eliminating these damaged organelles (76, 77).

In 2007, Ted Dawson and Valina Dawson from Johns Hopkins University School of Medicine discovered a new form of programmed brain cell death called Parthanatos, also known as poly ADP-ribose polymerase-1 (PARP-1) dependent cell death. Parthanatos is a type of programmed cell death that occurs due to DNA damage and the activation of PARP-1 (78). After spinal cord injury (SCI), there is an increase in oxidative stress which leads to the production of large amounts of ROS. These ROS molecules play a crucial role in activating Parthanatos (79, 80). Zinc is an essential nutrient that binds with various enzymes and transcription factors (81, 82). The neuroprotective effect of Zinc has been extensively studied (51, 83). Sirtuin 3 (SIRT3), a major mitochondrial acetyl-lysine deacetylase, is responsible for regulating mitochondrial homeostasis and ROS production (52). Recent findings suggest that Zinc acts on SIRT3 to prevent SCI by directly eliminating ROS through acetylation, targeting SOD2 to inhibit Parthanatos. Additionally, Zinc promotes mitophagy, indirectly eliminating ROS and alleviating Parthanatos. In summary, Zinc mediates anti-oxidative stress through SIRT3, reducing the expression of PARP-1 (84). This discovery reinforces the understanding of how Zinc protects against SCI by scavenging ROS.

**Figure 2:**
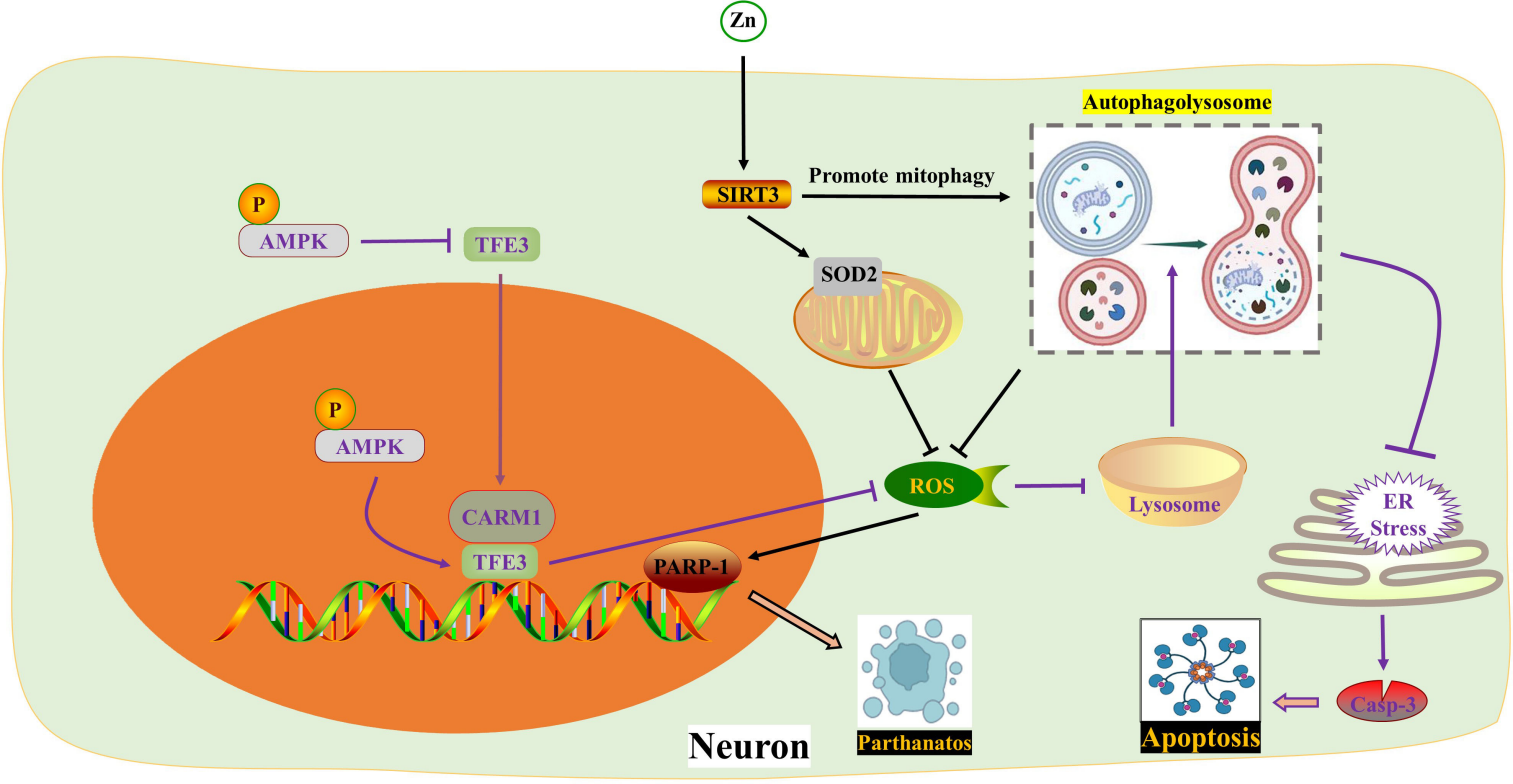
ROS in autophagy. Zinc interacts with SIRT3 to inhibit PARP-1-mediated Parthanatos by targeting mitochondrial SOD2 and promoting mitophagy to eliminate ROS respectively. TFE3 reduces ROS formation and enhances lysosomal activity to restore autophagy, thereby impeding endoplasmic reticulum stress and apoptosis. The activity of TFE3 is regulated by p-AMPK in both the cytoplasm and nucleus. AMPK, Adenosine 5’-monophosphate (AMP)-activated protein kinase; ER, Endoplasmic reticulum; PARP-1, poly ADP-ribose polymerase-1; ROS, Reactive oxygen species; SIRT3, Sirtuin 3; SOD2, Superoxide dismutase 2; TFE3, Transcription factor E3; Zn, zinc.

As mentioned above, the fusion of autophagosomes and lysosomes plays a crucial role in autophagy. In a study by Kailiang Zhou et al. (54) it was discovered that the excessive accumulation of ROS caused lysosomal dysfunction and blocked autophagic flux in SCI. This inhibition of autophagy then led to endoplasmic reticulum stress-dependent neuronal apoptosis. The researchers further confirmed these findings in neurons from ATG5^-/+^ mice. They also found that the transcription factor E3, which is partially regulated by the AMPK-mTOR and AMPK-SKP2-CARM1 signaling pathways, promoted autophagy-lysosomal activity and inhibited ROS accumulation. This restoration of autophagic flux after SCI helped alleviate neuronal damage. Interestingly, under SCI stimulation, AMPK phosphorylation in the cytoplasm inhibited the level of m-TOR and the nuclear translocation of TFE3. However, p-AMPK in the nucleus increased the levels of CARM1, which binds to TFEB to induce TFEB transcription (**Figure 2**) (**Table 1**).

Autophagy is a complex self-degradation process that involves multiple steps. In current literature, the assessment of autophagy levels often relies on the detection of autophagosomes or the expression of autophagy proteins. However, these measures may not provide a comprehensive evaluation of the smoothness of autophagic flow. It is important to note that a decrease in the degradation rate of autophagy substrates, caused by reduced lysosomal function due to endoplasmic reticulum stress, can weaken the overall autophagy process. This weakening can lead to an accumulation of autophagosomes and increased expression of autophagy proteins (85, 86). Electron microscopy is considered the gold standard for observing autophagy as it can capture both the induction of autophagy and the consumption of autophagosomes. Autophagy generally plays a protective role in cells, but disruption of the autophagy mechanism or excessive autophagy flux can result in cell death, known as autophagy-dependent cell death or autophagy-mediated cell death (80, 87, 88). Restoring lysosomal function and blocking autophagy flux can protect cells from apoptosis (89). Interestingly, blocking autophagic flux may also contribute to cell death or apoptosis (90). In oncology research, candesartan has been found to induce cell death in trail-resistant lung cancer cells by blocking autophagy flux (91). Taken together, regulating autophagic flux to protect body cells from damage or promote tumor death is a challenging task.

## 4 ROS in pyroptosis

The hallmark event of pyroptosis is the formation of pores in the cytoplasmic membrane by the Gasdermin (GSDM) protein family, primarily GSDMD and GSDME, which are cleaved by caspases. When exposed to external stimuli, inflammasomes recognize pathogen-related molecular patterns (PAMPs) or host-derived danger signal molecules (DAMPs) to activate caspase-1 (53, 92). In the classic pyroptosis pathway, inflammasomes such as NLRP1, NLRP2, NLRP3, NLRC4, and AIM2 dissociate and activate caspase-1. Activated caspase-1 has two main functions: cleaving GSDMD into GSDMD-NT to form membrane pores, and promoting the maturation of pro-IL-1β/18 into IL-1β/18, which is released from the GSDMD-NT pores, thereby amplifying the inflammatory response. In the non-classical pyroptosis pathway, external bacteria or viruses activate caspase-11 (in mice) or caspase-4/5 (in humans) through lipopolysaccharide (LPS) to cleave GSDMD and induce pyroptosis (93, 94). The GSDM family includes GSDM-A/B/C/D/E and DFNB59. However, DFNB59 does not form membrane pores through the previously mentioned pathways. The NLRP3 inflammasome, which consists of NLRP3, apoptosis-associated speck-like protein containing a caspase recruitment domain (ASC), and caspase-1, is the most extensively studied. Studies have shown that NLRP3 is activated and plays a crucial role in spinal cord injury (SCI) in various cell types, including neurons, microglia, astrocytes, and pericytes (94). Oxidative stress and the production of reactive oxygen species (ROS) are critical in the activation of the NLRP3 inflammasome and the initiation of pyroptosis. Currently, the main mechanisms of NLRP3 inflammasome activation include ROS formation, lysosomal rupture, and ion channel gating. Among these mechanisms, NADPH oxidase (NOX) is considered the most important trigger, mediating ROS generation (95, 96).

MAPKs, such as ERK1/2, JNK, and p38, are a group of highly conserved serine/threonine protein kinases that play a role in various processes including stress, inflammation, proliferation, differentiation, survival, apoptosis, and more. Previous studies have shown that MAPKs and ROS mutually affect each other’s activities (97). ROS can enhance the inflammatory response by activating multiple pro-inflammatory signaling pathways, such as MAPK, NF-κB, and JAK-STAT 9-12 (98). Additionally, the hyperphosphorylation of MAPKs amplifies subsequent inflammatory responses by activating NF-κB (99). Recent studies by Zhongyuan Liu and colleagues have confirmed that both LPS+ATP (55) and AOPPs (Advanced oxidation protein products) (92) activate BV2 cells through the ROS-dependent MAPKs-NF-κB signaling pathway, leading to NLRP3 inflammasome-mediated pyroptosis. Specifically, this process activates p-p38 and p-JNK MAPKs, but not p-ERK1/2. The natural polyphenol kaempferol and the selective NADPH oxidase inhibitor apocynin both inhibit the translocation of NF-κB p65 subunit into the nucleus and alleviate neuroinflammation by inhibiting the aforementioned pathways. In the upstream signaling pathway, the NADPH oxidase family is the primary source of ROS in BV2 cells (100). NOX2 (NADPH oxidase 2) and NOX4 are mainly expressed in spinal cord microglia. Interestingly, the authors found that kaempferol and AOPPs only regulate the expression of NOX4.

The voltage-gated proton channel (Hv1), a unique member of the superfamily of voltage-gated cation channels, facilitates the production of ROS (101, 102). Ion channels, including Hv1, play a crucial role in the physiological functions of neurons and glial cells in the nervous system (57, 103). Hv1 is a selective ion channel for H+ and plays a key role in regulating nicotinamide adenine dinucleotide phosphate NOX activity and pH in phagocytes (104). Hv1-mediated ROS production contributes to brain damage after cerebral ischemia (105). In microglia, Hv1 accelerates ROS generation by inducing NOX (59). A recent study revealed that Hv1 deficiency reduced microglial ROS production during SCI, leading to inhibition of neuronal apoptosis and NLRP3-mediated pyroptosis. *Hv1^−/−^* mice showed improved axonal regeneration and motor function recovery after SCI (106). Interestingly, this study found that the peak of neuronal apoptosis occurred earlier than neuronal pyroptosis, while the duration of pyroptosis was longer than that of apoptosis. However, the researchers did not provide further insights into the crosstalk between pyroptosis and apoptosis, which warrants further investigation.

The role of autophagy after SCI is a topic of debate among scholars. Many believe that moderate activation of autophagy helps nerve cells adapt and survive (58). Recent studies have shown that increased autophagy can reduce inflammation and pyroptosis (107, 108). In the context of alleviating SCI, betulinic acid (BA) and piperine have been found to activate autophagy, leading to a decrease in ROS formation and NLRP3/GSDMD-mediated pyroptosis (75, 109). Specifically, BA activates macroautophagy through the AMPK-mTOR-TFEB pathway, which in turn reduces ROS accumulation by enhancing mitophagy, thereby inhibiting pyroptosis (75). The interaction between autophagy and pyroptosis is complex, with ROS and mitochondrial homeostasis playing a crucial role in their communication.

**Figure 3:**
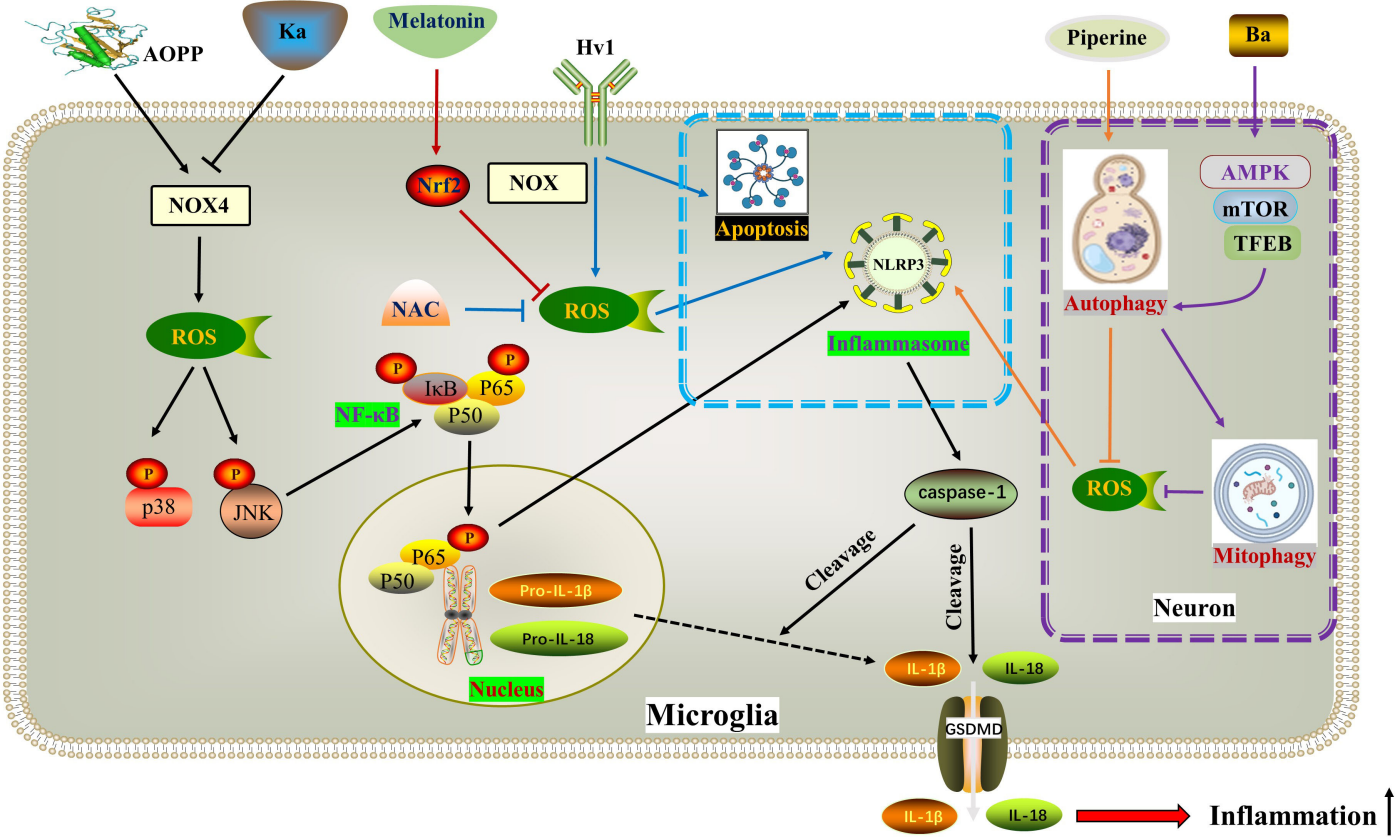
ROS in pyroptosis. AOPP increases NOX4-mediated ROS production, thereby phosphorylating and activating p38 and JNK as well as downstream NF-κB, ultimately activating NLRP3-dependent pyroptosis. Ka exhibits opposite effects through the same pathway. Hv1 accelerates neuronal NLRP3-mediated pyroptosis and apoptosis by promoting ROS production in microglia. Piperine reduces ROS formation and NLRP3/GSDMD-mediated pyroptosis by activating autophagy. BA activates macroautophagy through the AMPK-mTOR-TFEB pathway, thereby reducing ROS accumulation by enhancing mitophagy, thereby inhibiting pyroptosis. Melatonin inhibits the generation of ROS and the activation of NLRP3 inflammasome by activating the Nrf2/ARE signaling pathway. ^*^ The blue dotted box indicates the effect of Hv1-mediated microglial ROS production on neuronal pyroptosis and apoptosis. The purple dotted box shows autophagy and pyroptosis in neurons. AOPPs, Advanced oxidation protein products; BA, Betulinic acid; Hv1, The microglial voltagegated proton channel; Ka, Kaempferol; NOX4, Nicotinamide adenine dinucleotide phosphate hydrogen (NADPH) oxidases.

The Nrf2/ARE pathway is a major target for neuroprotection due to its anti-inflammatory and antioxidant effects (110, 111). Melatonin, a hormone primarily synthesized in the pineal gland that delays aging, resists oxidation, and improves sleep, is considered a positive regulator of Nrf2 (112–114). A study on SCI confirmed that Melatonin inhibited the NLRP3 inflammasome by activating Nrf2/ARE signaling. This resulted in the downregulation of inflammatory factors and reduced levels of reactive oxygen species (ROS), malondialdehyde, and improved mitochondrial function in neurons (115) (**Figure 3**) (**Table 1**).

## 5 ROS in ferroptosis

The concept of ferroptosis, which was first proposed in 2012 (116), is a novel model of regulated cell death (RCD) characterized by specific changes in cell morphology, such as mitochondrial atrophy and reduction or disappearance of mitochondrial cristae (117). Ferroptosis is a process in which the accumulation of lethal lipid reactive oxygen species (ROS) is dependent on iron and involves uninhibited lipid peroxidation induced by iron metabolism. Iron homeostasis in the nervous system is tightly regulated by various ferritins, including iron regulatory proteins (IRPs), divalent metal transporter 1 (DMT1), transferrin receptors (TfRs), ferritin, and iron transporter 1 (Fpn1) (118). Excessive iron in cells leads to mitochondrial damage and the generation of excessive ROS through the Fenton reaction, which reacts with polyunsaturated fatty acids (PUFAs) in the lipid membrane, causing lethal lipid peroxidation (119–121). The extrinsic pathway of ferroptosis involves the inhibition of cell membrane transport proteins or the activation of proteins involved in iron ion transport, while the intrinsic pathway involves the inhibition of intracellular antioxidant enzyme systems (122). For example, reduced activity of the antioxidant enzyme GPx4 fails to suppress intracellular ROS production, resulting in lipid peroxidation (116). Further research has identified several genes, including Ptgs2, Gpx4, and Fsp1, that regulate ferroptosis. Ptgs2 encodes cyclooxygenase-2 (COX-2) and is significantly upregulated during ferroptosis (123).

In the acute phase of spinal cord injury (SCI), the blood-spinal cord barrier and blood vessels are destroyed, resulting in an increase in iron concentration at the site of injury. This leads to a vicious positive cycle of iron overload, excessive production of reactive oxygen species (ROS), and ferroptosis, which worsens the loss of neurological function after SCI. Therefore, reducing iron overload is crucial in breaking this harmful pattern (124, 125). Current evidence suggests that several drugs show potential for the treatment of SCI, including the iron chelator deferoxamine, the ferroptosis agent SRS 16-86, zinc and proanthocyanidins, and the Nrf2 pathway activator carnosic acid (60, 126–129). Interestingly, drug delivery to the central nervous system has been hindered by the blood-brain barrier and blood-spinal cord barrier. Some studies have found that oral iron chelators, like deferiferrin, have limited or no effect on improving motor function after SCI (61). However, there are iron chelators like hydroxybenzylethylenediamine (HBED) that can cross the blood-brain barrier, and minocycline, an antibiotic with iron-chelating properties. Both of these substances can alleviate neurological damage by reducing microglial activation (130, 131).

Motor neuron death following spinal cord injury (SCI) can result in atrophy of the primary motor cortex. Zhou Feng et al. (123) conducted a study which revealed a significant increase in iron deposition in motor cortex neurons of both SCI patients and rats. This iron deposition triggered the accumulation of ROS and led to neuronal ferroptosis. However, the researchers found that the use of an iron chelator called DFO, a ROS inhibitor called NAC, and a ferroptosis inhibitor called Fer-1 successfully reversed neuronal ferroptosis. Further investigations indicated that the release of NO from activated microglia after SCI resulted in an increased expression of IRP1, DMT1, and TfR1, while ferritin expression decreased. The inhibition of microglial activation by minocycline and the inhibition of NO synthase by L-NAME were both found to reverse neuronal ferroptosis and promote the recovery of motor nerve function. Therefore, therapeutic approaches aimed at balancing iron metabolism hold promise for the treatment of SCI.

Trehalose, a non-reducing disaccharide with antioxidant effects, has shown promise in improving the prognosis of various neurological diseases, including Huntington’s disease, Alzheimer’s disease, and amyotrophic lateral sclerosis (62, 63, 132). In a study by Fangyi Gong et al. (133), it was found that trehalose effectively reduced oxidative stress and ROS generation after spinal cord injury (SCI). This reduction was accompanied by an increase in GSH and Gpx4 content, as well as a decrease in MDA and Acsl4 levels, neuronal degeneration, and iron accumulation. The mechanism behind this effect involves the activation of the NRF2/HO-1 signaling pathway, which inhibits neuronal cell ferroptosis. Additionally, trehalose has been found to reduce neuroinflammation after SCI, possibly by suppressing ROS and subsequent ferroptosis.

**Figure 4:**
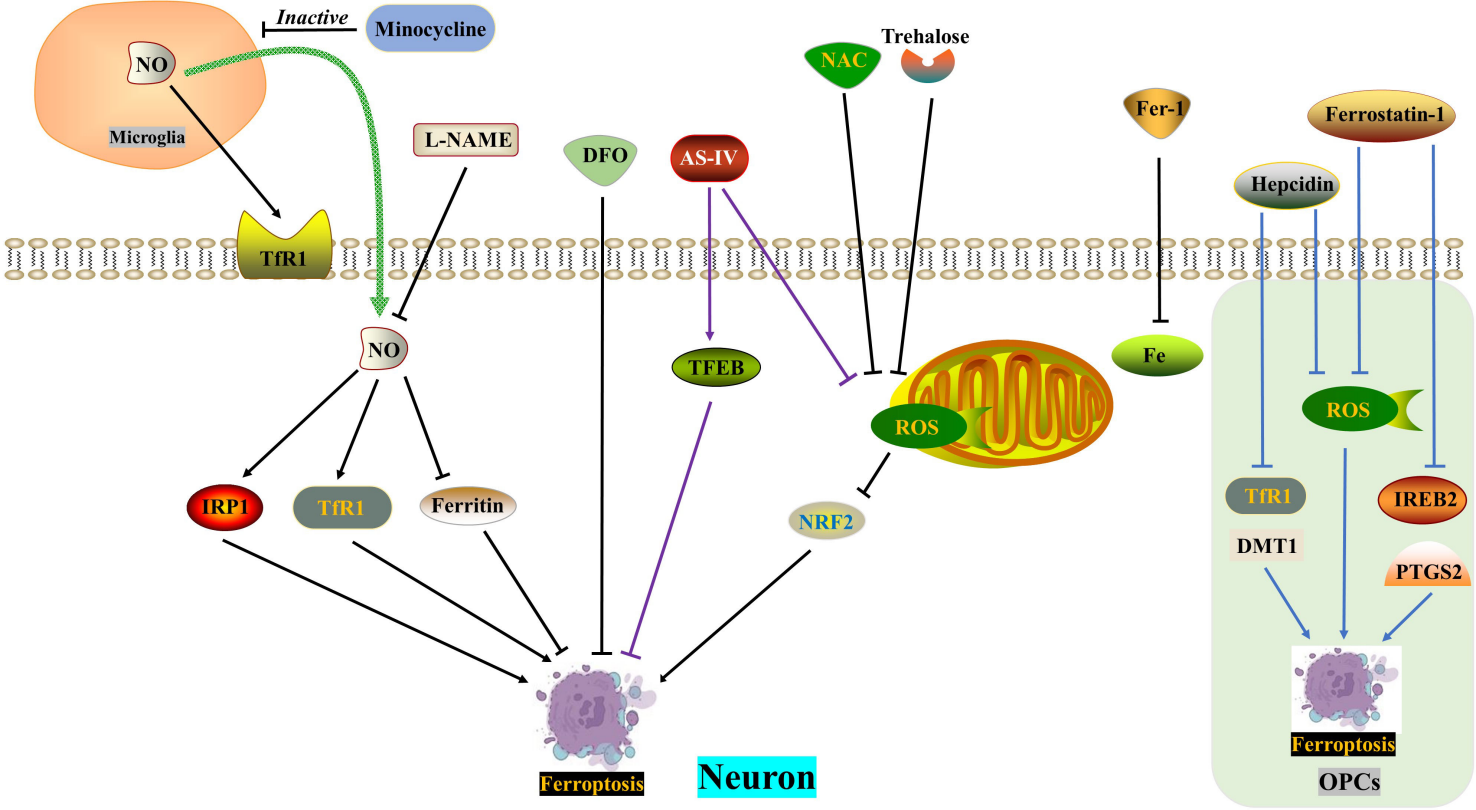
ROS in ferroptosis. DFO, NAC and Fer-1 reverse SCI-induced neuronal ferroptosis, respectively. SCI induces increased NO release from microglia and exacerbates neuronal ferroptosis, which is reversed by minocycline and L-NAME. Trehalose inhibits neuronal ferroptosis through the ROS/NRF2 pathway. AS-IV alleviates neuronal ferroptosis by inhibiting ROS generation and promoting TFEB expression. Hepcidin ameliorates ferroptosis in OPCs by repressing ROS level and DMT1/TfR1 expression. Ferrostatin-1 reduces ferroptosis in OPCs by inhibiting ROS generation and IREB2/PTGS2 level. AS-IV, Astragaloside IV; DFO, Deferoxamine, an iron chelator; DMT1, Divalent metal transporter 1; FAC, Ferric ammonium citrate; Fer-1, Ferrostatin-1, a ferroptosis inhibitor; IRP1, Iron regulatory protein 1; L-NAME, an NO synthase inhibitor; NAC, N-acetylcysteine, a ROS inhibitor; NO, Nitric oxide; OPCs, oligodendrocyte progenitor cells; ROS, Reactive oxygen species; TfR1, Ransferrin receptor1.

TFEB, a member of the microphthalmia-associated transcription factor family, plays a crucial role in the oxidative stress process (134, 135). In a study, Astragaloside IV (AS-IV) was found to mitigate H2O2-induced damage to PC12 cells by inhibiting the generation of ROS and promoting TFEB expression, thereby preventing ferroptosis. The protective effects of AS-IV were reversed when TFEB was knocked down (136). The integrity of white matter bundles is vital for motor function recovery after SCI (137), as well as for neuronal communication and cognitive function improvement (138). Oligodendrocytes, which wrap around axons, form the basic structure of white matter bundles. Due to their high content of unsaturated fatty acids, oligodendrocytes are vulnerable to iron toxicity. Research by Shengli Hu demonstrated that both hepcidin and ferrostatin-1 can inhibit ferroptosis in OPCs by reducing ROS production. However, they differ in their mechanisms: hepcidin down-regulates the expression of DMT1 and TfR1, while ferrostatin-1 negatively modulates the expression of IREB2 and PTGS2 (139, 140). Hepcidin, a peptide hormone primarily secreted by the liver, can also reduce iron uptake and release by astrocytes and neurons by decreasing the expression of Fpn1, TfR1, and DMT1 (141–143) (**Figure 4**) (**Table 1**).

## 6 ROS removal in materials science development

In order to effectively reduce oxidative stress and eliminate excess ROS, researchers are focusing on developing new carriers or materials such as exosomes, hydrogels, and nanomaterials. Exosomes are nanoscale extracellular vesicles with a particle size of 50-150nm. They play a paracrine role by transmitting genetic material information, including non-coding RNA, mRNA, and proteins. Due to their nanometer size and membrane permeability, exosomes are considered a promising approach for treating central nervous system diseases (144–146). One study showed that the application of exosomes derived from microvascular endothelial cells (USP13) alleviated SCI injury by improving mitochondrial damage and reducing ROS production (147). Hydrogels, which have good biocompatibility, hydrophilicity, and controlled drug release, also have unique advantages in SCI treatment. However, their use for SCI treatment is limited due to the high fragility of the spinal cord, as hydrogels with high hardness cannot be used. Yibo Ying et al. (148) utilized TPA and Laponite to develop hydrogels that exhibit shear thinning capabilities. They conducted experiments to demonstrate that these hydrogels can synergistically impede ferroptosis by enhancing vascular function and regulating iron metabolism. Additionally, some researchers have successfully prepared a polylipoic acid-sodium lipoate hydrogel (PLAS) with desirable mechanical properties, easy injectability, and sufficient adhesion. Animal experiments have revealed that PLAS can effectively eliminate cellular ROS, thereby promoting recovery from spinal cord injury (149).

Nanoparticles offer significant advantages in terms of size manipulation, chemical and physical properties. Researchers often modify the size and surface of nanoparticles to minimize biological clearance, evade immune defense systems, prolong the duration of action, and enhance drug release. Selenium nanoparticles have gained significant attention as potential carriers for antioxidant drugs. In a study by Siyuan Rao et al. (150), a multifunctional functionalized selenium nanoparticle loaded with tetramethylpyrazine (TMP)/monosialotetrahexosylganglioside (GM1) (SeNPs@GM1/TMP) was designed and synthesized. The researchers found that SeNPs@GM1/TMP effectively reduced the excessive production of ROS by inhibiting the activation of p53 and MAPKs pathways, thereby preventing mitochondrial dysfunction and promoting functional recovery after SCI. Another experiment conducted by the same research team involved loading astragalus polysaccharides (APS) and tanshinone IIA (TSIIA) on SeNPs (TSIIA@SeNPs-APS), which also demonstrated effective ROS inhibition and protection of neuronal cells (151). Additionally, a chitosan-modified hollow manganese dioxide nanoparticle system loaded with resveratrol (RESV) was found to effectively reduce ROS, MDA, and SOD levels, while increasing glutathione (GSH) levels (152). This reduction in inflammation and neuronal apoptosis after SCI was observed. Similarly, loading RES into plasma complex component functionalized manganese-doped silica nanoparticles (PMMSN) carrier (PMMSN-RES) also led to reduced ROS, MDA, and inflammatory factors (TNF-α, IL-1β, and IL-6) after SCI, thereby promoting neuronal survival and restoring spinal cord functionality in mice (153). In another study by Haifei Shi et al. (154), the mesoporous pores and hydrophobic benzene ring structure of polydopamine (mPDA) were utilized to effectively encapsulate and sustainably release rapamycin (Rapa) (mPDA@Rapa). *In vitro* experiments demonstrated that these nanoparticles inhibited excessive ROS production and reduced spinal cord inflammation. Furthermore, some scholars have developed a highly biologically active iridium complex (IrFPHtz) that can directly target SOD1, upregulating its expression and eliminating excessive ROS production induced by SCI (155). The continuous advancement of nanomaterial technology allows for ongoing exploration of the ROS scavenging ability and antioxidant effect of nanomaterials in the treatment of spinal cord injury (151, 156, 157). These innovative materials hold great potential for the treatment of SCI and may become promising candidates for clinical practice in the future.

## 7 Conclusions and future perspectives

SCI imposes a significant financial burden on patients and caregivers, with estimated lifetime care costs ranging from $1.6 million to $4.8 million. It is widely accepted that inflammation plays a role in the progression of SCI and subsequent neurological dysfunction. ROS and RNS are early events in mechanical damage in SCI. The excessive production of ROS, such as •OH, H2O2, and •O2−, during secondary injury surpasses the body’s antioxidant capacity, leading to increased oxidative stress and regulated cell death (RCD). Excessive activation of iNOS results in the overproduction of NO, which can react with •O^2−^ to form peroxynitrite (PN: ONOO−), further promoting DNA and lipid peroxidation (158). The cascade of oxygen radical reactions begins with the production of O_2_•^−^, which reacts with nitric oxide (•NO) radicals to form the highly reactive oxidant peroxynitrite (PN: ONOO−) and the by-product hydroxyl radical. SOD can quickly catalyze the decomposition of O_2_•^−^ into H_2_O_2_ and oxygen. Subsequently, PN decomposition leads to the formation of highly reactive cytotoxic free radicals, including nitrogen dioxide (•NO_2_) and carbonate radicals (•CO_3_). These PN-derived free radicals induce oxidative damage to proteins, lipids, and nucleic acids (159, 160), impairing mitochondrial respiration and Ca^2+^ buffering capacity (161). Additionally, PN products can stimulate lipid peroxidation. The formation of peroxynitrite and lipid peroxidation irreversibly impair neuronal membrane lipid and protein function, leading to subsequent ion homeostasis imbalance, glutamate-mediated excitotoxicity, mitochondrial respiratory failure, and microvascular damage (7). Therefore, compounds that inhibit the formation of ROS and RNS or scavenge ROS and RNS are effective strategies for the treatment of SCI.

Currently, there is no FDA-approved strategy for effectively improving SCI. Given the detrimental impact of oxidative stress and inflammation on SCI progression, reducing ROS levels and oxidative stress is generally considered a fundamental treatment approach (162). As mentioned earlier, SCI triggers various forms of RCD, such as apoptosis, autophagy, pyroptosis, and ferroptosis, due to excessive accumulation of free radicals, mitochondrial dysfunction, glutamate release, and calcium overload. There is intricate crosstalk between signaling pathways involved in these types of RCD. Considering the pivotal role of ROS in the inflammatory cascade following SCI, antioxidants have emerged as a key area of research for scavenging ROS, reducing oxidative stress, and alleviating RCD. Endogenous antioxidants include SOD, CAT, GPx-1, thiol antioxidants (glutathione, α-lipoic acid), uric acid, and coenzyme Q10 (CoQ10) (163, 164). Exogenous antioxidants, including vitamin C (ascorbic acid), vitamin A, carotenoids, and polyphenols have promising applications in central nervous system trauma and neurological degenerative diseases by regulating RCD of nerve cells and glial cells (165–169). Additionally, there are synthetic antioxidants like butylated hydroxyanisole, butylated hydroxytoluene, propyl gallate, and tert-butylhydroquinone. However, drug delivery challenges, particularly effectively penetrating the blood-spinal cord barrier and blood-brain barrier, and being taken up by target cells, are major concerns (170). The responsibility of weighing the benefit-risk balance of a drug lies with the clinician. Edaravone, a free radical scavenger, is currently the only FDA-approved antioxidant treatment drug, used for early-stage amyotrophic lateral sclerosis in the United States and Japan. However, its benefit is minimal in advanced cases, leading to the withdrawal of Radicava (the active substance of Edaravone) from marketing authorization in Europe (171). To overcome the limitations of natural and synthetic antioxidants, exosome therapy and new nanomaterials offer new possibilities for antioxidant treatment, providing more efficient drug delivery and greater antioxidant effects (172, 173). Cuproptosis, PANoptosome, and endoplasmic reticulum stress are also hot topics in this research field. In conclusion, the improvement of antioxidant therapy and the discovery of new antioxidants for alleviating ROS-induced RCD continue to provide more targets for SCI treatment.

## Author contributions

ZY: Data curation, Investigation, Methodology, Software, Writing – original draft, Writing – review & editing. BW: Writing – review & editing, Resources, Software, Visualization. GG: Investigation, Methodology, Project administration, Resources, Writing – review & editing. JY: Investigation, Methodology, Project administration, Resources, Software, Visualization, Writing – original draft, Writing – review & editing.
